# Metabolic response of *Gardenia jasminoides* cell suspensions to chitosan elicitation: bioactivity and phytochemical enrichment

**DOI:** 10.3389/fpls.2026.1807327

**Published:** 2026-05-14

**Authors:** Durga Anusha Sampath Kumar, Deepa Sankar Parasurama

**Affiliations:** 1 School of Bio Sciences and Technology, Vellore Institute of Technology, Vellore, Tamil Nadu, India; 2 Vellore Institute of Technology (VIT) School of Agricultural Innovations and Advanced Learning, Vellore Institute of Technology, Vellore, Tamil Nadu, India

**Keywords:** antibacterial, antioxidant, bioactive metabolites, chitosan, *Gardenia jasminoides*, suspension culture

## Abstract

**Introduction:**

*Gardenia jasminoides* Ellis is one of the most extensively investigated therapeutic plants and is a major source of bioactive phytoconstituents. Elicitor-mediated suspension cultures provide a transformative approach for bioactive metabolite enhancement to meet the escalating demand in the pharmaceutical sector. This study is the first to investigate the impacts of chitosan (CH)-elicited *G. jasminoides* suspension cultures on their bifunctional and metabolic profiling.

**Methods:**

The suspension cultures were obtained by employing optimal phytohormone regulators [0.5 mg/ L kinetin (KT) and 1 mg/L 2,4-dichlorophenoxyacetic acid (2,4-D)] in Murashige and Skoog (MS) medium, along with the application of CH elicitor. Antioxidant and antibacterial activities were evaluated, and metabolic profiling was performed using gas chromatography–mass spectrometry (GC-MS).

**Results:**

CH elicitation improved antioxidant capacity (28.93 ± 0.15%) with a fourfold increase, and marginal antibacterial activity was obtained at 40 mg/L CH. GC-MS assessment demonstrated crucial bioactive compounds [benzenamine, 2-(cyclopropylmethyl)-4,5- dimethoxy, *(E)*-3-(3,4-dimethoxyphenyl)-prop-2-enamide, methyl 8-methyldecanoate, N1-(4-hydroxybutyl)-N3-methylguanidine acetate, and trans-13- octadecenoic acid, methyl ester] of medicinal potential solely identified in the elicited extract.

**Discussion:**

This study emphasized the critical influence of CH-induced elicitation strategy on *G. jasminoides* in vitro cultures, elucidated specifically for the metabolic activation of elicitation-specific bioconstituents for forthcoming drug formulations.

## 1 Introduction

There is a vast diversity of biologically active compounds in plant resources that are used to produce medicinal molecules (Bergonzi et al., 2022). According to the World Health Organization (WHO), there is now 80% of the world’s population depending on folk medicinal traditions as a source of their phytomedicines (Canter et al., 2005; Kolewe et al., 2008; Sagharyan et al., 2020). The fusion of various biotechnological techniques has been observed to overcome the increasing need for bioactive components from plants. This is connected with an enigmatic full utilization of phytoplanktonic species (Salma et al., 2018).

Plant tissue culture (PTC) represents a promising biotechnological tool useful for micropropagation, habitat restoration, and genetic stock management. In addition, PTC aids in overcoming the hindrance of secondary metabolite production, which is limited by geographical distribution and climatic variability through *in vitro* culture development under specific conditions (Kumar and Parasurama, 2024a). *In vitro* callus and suspension cultures can be suitable systems for the efficient biosynthesis of commercially valuable bioactive secondary metabolites (Sák et al., 2014). In addition, the most important potential of *in vitro* cultures is their ability to improve secondary metabolite production via elicitor application. This elicitation strategy leads to the upregulation of defense-related genes and the production of enzymes, which in turn promotes the synthesis of bioactive compounds (e.g., phenolics, phytoalexins, flavonoids, terpenoids, and other bioactive metabolites) (Namdeo, 2007; Chavan et al., 2021). Biotechnological approaches have offered cell suspension cultures that can accumulate a large amount of secondary metabolites in reaction to diverse signaling molecules with biological (biotic) and chemical (abiotic) origin. This provides an efficient alternative for the enrichment of metabolites on a vast spectrum in a short period, which ameliorates issues of scarcity (Park et al., 2021; Kumar and Parasurama, 2024b).

Among the genus *Gardenia*, *Gardenia jasminoides* Ellis (cape jasmine) is a well-studied medicinal plant species. The leaves of *G. jasminoides* can act as a main reservoir of many bioactive compounds from the major groups, which are phenols, flavonoids, and terpenoids (Krasteva et al., 2022). These metabolites can act as potential targets for antioxidant and antimicrobial research in the pharmaceutical industry (Yoga et al., 2022; Bairagi et al., 2023). The increasing requirement for *G. jasminoides* secondary metabolites has also promoted other research efforts on PTC in it (Liu et al., 2018). Chitosan (CH), a partially deacetylated polysaccharide derived from chitin, functions as an effective biotic elicitor by simulating pathogen-associated molecular signals, thereby initiating the plant defense responses. In contrast to many synthetic elicitors, CH is biocompatible, biodegradable, and non-toxic, supporting its application as an eco-friendly agent for stimulating the biosynthesis of secondary metabolites. In addition to its biological role, CH possesses favorable physicochemical properties, such as solubility in dilute acidic solutions and suitability for application in scalable culture systems. CH promotes the activation of phenylpropanoid metabolism and enhances the accumulation of phenolic and flavonoid compounds, offering a reliable and sustainable approach for improving the production of valuable phytochemicals in *in vitro* cell cultures (Kumar and Parasurama, 2025).

In our previous studies, the total yield of secondary metabolites was studied with two different elicitors [CH and methyl jasmonate (MJ)] in *G. jasminoides* suspension culture. CH elicitation treatment showed the highest total metabolite level (Kumar and Parasurama, 2025). However, there are no reports on the antioxidant activity, antibacterial effects, and pharmacological profiling of diversified bioactive metabolites in CH-elicited suspension cultures of *G. jasminoides*. As far as the existing literature, this study is the first to provide an overall view of the antioxidant activity, antibacterial potential, and bioactive metabolite profiling of CH-elicited *G. jasminoides* suspension cultures. Therefore, our findings provide an initial basis for subsequent scientific studies on increased metabolite accumulations contributing to antioxidant activity and antibacterial ability.

## 2 Materials and methods

### 2.1 Suspension culture and elicitor treatment

*G. jasminoides* saplings obtained from Coimbatore, Tamil Nadu, India, were authenticated (voucher no. VITOD01-208) by the VIT Herbaria Vellore Institute of Technology, Vellore, Tamil Nadu, India. Young leaf bit explants were cultured using various plant growth regulator concentrations on Murashige and Skoog (MS) media and incubated (25 ± 2°C) in the dark to induce callus initiation. The effective treatment was sub-cultured at 4-week intervals to obtain *in vitro* friable calli for the initiation of suspension culture. The suspension culture of *G. jasminoides* was established by inoculating approximately 1 g of friable calli (sixth sub-culture) into MS media (80 ml) with 0.5 mg/L kinetin (KT) and 1 mg/L 2,4-dichlorophenoxyacetic acid (2,4-D) and cultured (25 ± 2°C) in a gyratory shaker (120 rpm) in the dark. The *in vitro* suspension culture was then subjected to elicitation using varying doses of CH and MJ (single and combined). The growth parameters and the total metabolite (phenol, flavonoid, and terpenoid) contents were subsequently analyzed (Kumar and Parasurama, 2025).

### 2.2 Sample extract preparation

Fresh biomass (2 g) from both unelicited and CH-elicited *in vitro* suspension cultures, harvested at their respective peak metabolite accumulation stages (phenols, 40 mg/L at 144 h; flavonoids, 40 mg/L at 120 h; and terpenoids, 80 mg/L at 36 h), was extracted with 10 ml of 80% methanol [gradient grade for HPLC, molecular weight (MW) = 32.04; Finar Chemicals, Ahmedabad, India) and incubated overnight on a shaker at 120 rpm under ambient conditions. The extracts were then sonicated for 15–20 min and centrifuged at 6,000 rpm for 5 min. The supernatant was collected and stored at −20°C for subsequent analysis, as described by El-Ashry et al. (2019).

### 2.3 Assessment of antioxidant potency

The antioxidant potential of the extracts obtained from both unelicited and CH-elicited *in vitro* suspension cultures was evaluated using the 2,2-diphenyl-1-picrylhydrazyl (DPPH) radical scavenging test, following the protocol described by Hendel et al. (2021), with minor modifications. Briefly, 1 ml of each sample extract at varying concentrations (25, 50, 75, and 100 µl/ml) was added to 2 ml of 0.1 mM DPPH solution. The reaction mixture was vortexed and incubated in the dark for 30–45 min at ambient temperature. The absorbance was measured at 517 nm using an ultraviolet spectrophotometer. The radical scavenging activity was calculated as percentage inhibition using the formula: DPPH inhibition (%) = (CA − SA/CA) × 100, where CA represents the control absorbance and SA represents the sample absorbance.

### 2.4 Assessment of antibacterial potency

The antibacterial potency of the culture extracts (non-elicited and CH-elicited suspension cultures) was assessed utilizing the agar well diffusion assay. Each sample extract was formulated at four distinct concentrations (25–100 µl/ml). The antibacterial efficacy was investigated using Gram-negative (*Pseudomonas aeruginosa* ATCC 27853 and *Escherichia coli* ATCC 25922*)* and Gram-positive strains (*Staphylococcus aureus* ATCC 25923 and *Bacillus subtilis* ATCC 6633). An 18-h-old bacterial broth culture (using sterile swabs) was inoculated onto nutrient agar media. Four wells were perforated utilizing a cork borer (6 mm diameter). Different concentrations of the extracts were dispensed into the agar wells and then allowed to diffuse at ambient temperature. Bacterial culture Petri dishes were cultured for 18–24 h (at 37°C). The bacterial inhibition zones were evaluated employing a millimeter ruler (Saha et al., 2023).

### 2.5 GC-MS analysis

The extracts obtained in *Section 2.2* were reconstituted in 1 ml of HPLC-grade methanol and filtered through a 0.22-μm membrane filter. Sample extracts (1 µl) were injected through the injection system [Agilent 7693A (automated), Santa Clara, CA, USA] into the gas chromatography–mass spectrometry (GC-MS) instrumentation (model GC 8890/MS5977C). The injection volume represented a concentrated extract derived from a 1:5 (*w*/*v*) ratio of fresh biomass (unelicited and elicited cultures) to solvent (2 g of fresh cells extracted in 10 ml 80% methanol). An Agilent GC-MS with a capillary column (DB-5ms; length, 300 cm; internal diameter, 0.025 cm; and stationary phase film, 0.25 µm thickness) was employed. Ultrahigh-purity helium gas (carrier medium at 99.999%) was sustained at 1 ml/min flow rate. The thermal gradient programs were kept for 1 min in an oven (50°C/min and 10°C/min). The final temperature was sustained at 300°C (for 1 min). The port of injection was stabilized (250°C), and the GC-MS instrument was configured to detect ions from 0.6 to 1,091 *m*/*z*. Metabolites were identified utilizing the reference NIST20 spectral library through mass spectral analysis and retention indices. Data interpretation was performed utilizing the MassHunter software system (Frolova et al., 2025).

### 2.6 Statistical analysis

Experiments were tested in triplicate employing three independent *in vitro* culture samples (*n* = 3) and reported as the mean value ± standard error (SE). One-way analysis of variance (ANOVA) was utilized for statistical assessment. Subsequent analysis (*post hoc*) was carried out at the 5% significance level with IBM SPSS software (version 27) using Duncan’s multiple range test. ClustVis (a free online tool to visualize multivariate data clustering) was employed for principal component analysis (PCA) and hierarchical clustering heatmap (Metsalu and Vilo, 2015). IBM SPSS software (version 27) was utilized to perform Pearson’s correlation analysis.

## 3 Results

### 3.1 Determination of the antioxidant potency of CH-elicited suspension culture

The effects of optimal CH concentrations at particular time intervals [which resulted in maximum levels of phenol (40 mg/L CH at 144 h), flavonoid (40 mg/L CH at 120 h), and terpenoid (80 mg/L CH at 336 h] on the antioxidant activity were investigated. The antioxidant potency of the unelicited and elicited *G. jasminoides* suspensions was evaluated using DPPH inhibition analysis. The outcomes are presented in **Table 1**. The cell suspension cultures elicited with 40 mg/L CH at 144 h (100 µl/ml) exhibited the highest inhibition efficacy against DPPH, with a fourfold increase (28.93 ± 0.15) over the non-elicited culture (7.22 ± 0.27).

**Table 1:** Antioxidant activity of the unelicited and chitosan (CH)-elicited *Gardenia jasminoides in vitro* suspension cultures.

Antioxidant activity (inhibition, %)						
Extract concentration (µl/ml)	Control (36 h)	80 mg/L CH (36 h)	Control (120 h)	40 mg/L CH (120 h)	Control (144 h)	40 mg/L CH (144 h)
25	0.78 ± 0.12**c**	0.85 ± 0.22**c**	2.28 ± 0.07**b**	20.13 ± 0.25**a**	2 ± 0.18**b**	20.38 ± 0.18**a**
50	1 ± 0.09**f**	2 ± 0.21**e**	4.07 ± 0.22**d**	22.38 ± 0.21**b**	4.72 ± 0.18**c**	23.78 ± 0.19**a**
75	3.25 ± 0.18**d**	3.5 ± 0.27**d**	5.07 ± 0.27**c**	24.17 ± 0.23**b**	5.72 ± 0.15**c**	26.71 ± 0.18**a**
100	4.39 ± 0.16**e**	6.86 ± 0.22**cd**	6.43 ± 0.22**d**	26.68 ± 0.18**b**	7.22 ± 0.27**c**	28.93 ± 0.15**a**

The experiment was performed in triplicate. Statistical analysis was conducted using IBM SPSS Statistics (version 27.0), applying Duncan's multiple range test at a significance threshold of P ≤ 0.05. For each variable, means at each concentration with the same letter indicate no significant difference compared to the preceding lower concentration in descending order.

Data are presented as the mean ± standard error (SE). Experiments were performed in triplicate (*n* = 3) using three independent biological batches of cell suspension cultures to account for inter-batch variability. Statistical evaluation was performed using one-way analysis of variance (ANOVA) followed by Duncan’s multiple range test at a 5% significance level (*p* < 0.05). Means within a column followed by identical letters are not significantly different. Independent biological replicates were defined as separate flasks initiated from distinct subculture cycles.

The results showed that treatment with CH at 40 mg/L for 120 and 144 h had the most significantly effective DPPH inhibition relative to treatment with CH at 80 mg/L for 36 h. The increase in efficacy may be due to the high levels of flavonoids and phenols present in CH-induced cultures at a concentration of 40 mg/L at 120 and 144 h (Kumar and Parasurama, 2025). These findings highlight that the use of the DPPH assay in association with phenol and flavonoid quantification is a reliable method for the determination of antioxidant power in elicited cultures. Our prior study reported that *G. jasminoides* callus tissue exhibited minimal antioxidant activity compared with the *in vitro* suspension cultures at maximum biomass, with improved levels of secondary metabolites (Kumar and Parasurama, 2024a). In this study, the results demonstrated that optimized dose and time determined for CH elicitation notably elevated the metabolite synthesis, with a 6.8-fold increase over callus cultures and a 1.8-fold increase over suspension cultures at the peak biomass.

### 3.2 Determination of the antibacterial potency of CH-elicited suspension culture

Inhibition zones were assessed using four bacterial microorganisms (*P. aeruginosa*, *E. coli*, *B. subtilis*, and *S. aureus*) via an agar well diffusion assay for sample extracts with varying concentrations (25–100 µl/ml) from optimal CH-elicited and non-elicited cell suspension cultures of *G. jasminoides*, as presented in **Table 2**. These microorganisms were selected to represent a broad spectrum of clinically relevant pathogens, aligning with the traditional use of *G. jasminoides* in folk medicine for the management of conditions such as wound infections and enteric disorders owing to its reported antimicrobial, anti-inflammatory, and antipyretic properties (Yoga et al., 2022). CH at 40 mg/L with 120- and 144-h exposure periods at a concentration of 100 µl/ml demonstrated antibacterial efficacy by producing inhibition zones against all four tested microorganisms, whereas no inhibition zones were observed in the non-elicited control suspension cultures. Nonetheless, the extract of CH at 80 mg/L, with a 36-h exposure period at a concentration of 100 µl/ml, demonstrated selective antibacterial efficacy, evidenced by the inhibition zones against *E. coli* and *P. aeruginosa*, while showing no inhibitory effects against *B. subtilis* and *S. aureus*. The antibacterial efficacy of the CH-elicited extracts was quantitatively compared with that of the standard antibiotic erythromycin. While the extracts produced measurable inhibition zones, they remained significantly smaller (6.13–7.33 mm) than those of erythromycin, which ranged from 9 to 17 mm across the four tested strains. This indicates that while CH elicitation successfully triggers the synthesis of antimicrobial secondary metabolites, their potency is moderate to weak compared with that of commercial standards.

**Table 2:** Antibacterial potency of non-elicited and chitosan (CH)-elicited *Gardenia jasminoides* suspension cultures.

Antibacterial potency (inhibition zone, mm)								
Bacterial strain	Extract concentration (µl/ml)	Positive control (erythromycin)	Non-elicited suspension culture			Elicited suspension culture		
			Control			80 mg/L CH	40 mg/L CH	40 mg/L CH
			36 h	120 h	144 h	36 h	120 h	144 h
*Bacillus subtilis*	25	9	NIR	NIR	NIR	NIR	NIR	NIR
	50	10	NIR	NIR	NIR	NIR	(6.33 ± 0.16)	NIR
	75	12	NIR	NIR	NIR	NIR	(6.66 ± 0.16)	(6.4 ± 0.1)
	100	14	NIR	NIR	NIR	NIR	(6.66 ± 0.16)	(6.56 ± 0.06)
*Staphylococcus aureus*	25	13	NIR	NIR	NIR	NIR	NIR	NIR
	50	14	NIR	NIR	NIR	NIR	(6.4 ± 0.05)	NIR
	75	16	NIR	NIR	NIR	NIR	(6.46 ± 0.08)	NIR
	100	17	NIR	NIR	NIR	NIR	(6.93 ± 0.06)	(6.43 ± 0.03)
*Escherichia coli*	25	9	NIR	NIR	NIR	NIR	NIR	NIR
	50	10	NIR	NIR	NIR	NIR	NIR	NIR
	75	11	NIR	NIR	NIR	NIR	(6.66 ± 0.16)	NIR
	100	12	NIR	NIR	NIR	(6.66 ± 0.16)	(7.33 ± 0.33)	(6.83 ± 0.16)
*Pseudomonas aeruginosa*	25	9	NIR	NIR	NIR	NIR	NIR	NIR
	50	11	NIR	NIR	NIR	NIR	NIR	NIR
	75	12	NIR	NIR	NIR	NIR	NIR	NIR
	100	13	NIR	NIR	NIR	(6.13 ± 0.03)	(6.4 ± 0.05)	(6.2 ± 0.05)

Data are presented as the mean ± standard error (SE). Experiments were performed in triplicate (*n* = 3) using three independent biological batches of cell suspension cultures to account for inter-batch variability. Independent biological replicates were defined as separate flasks initiated from distinct subculture cycles.

*NIR*, no inhibitory response

### 3.3 Metabolite analysis of unelicited and CH-elicited *G. jasminoides* suspension cultures via GC-MS profiling

**Figure 1:**
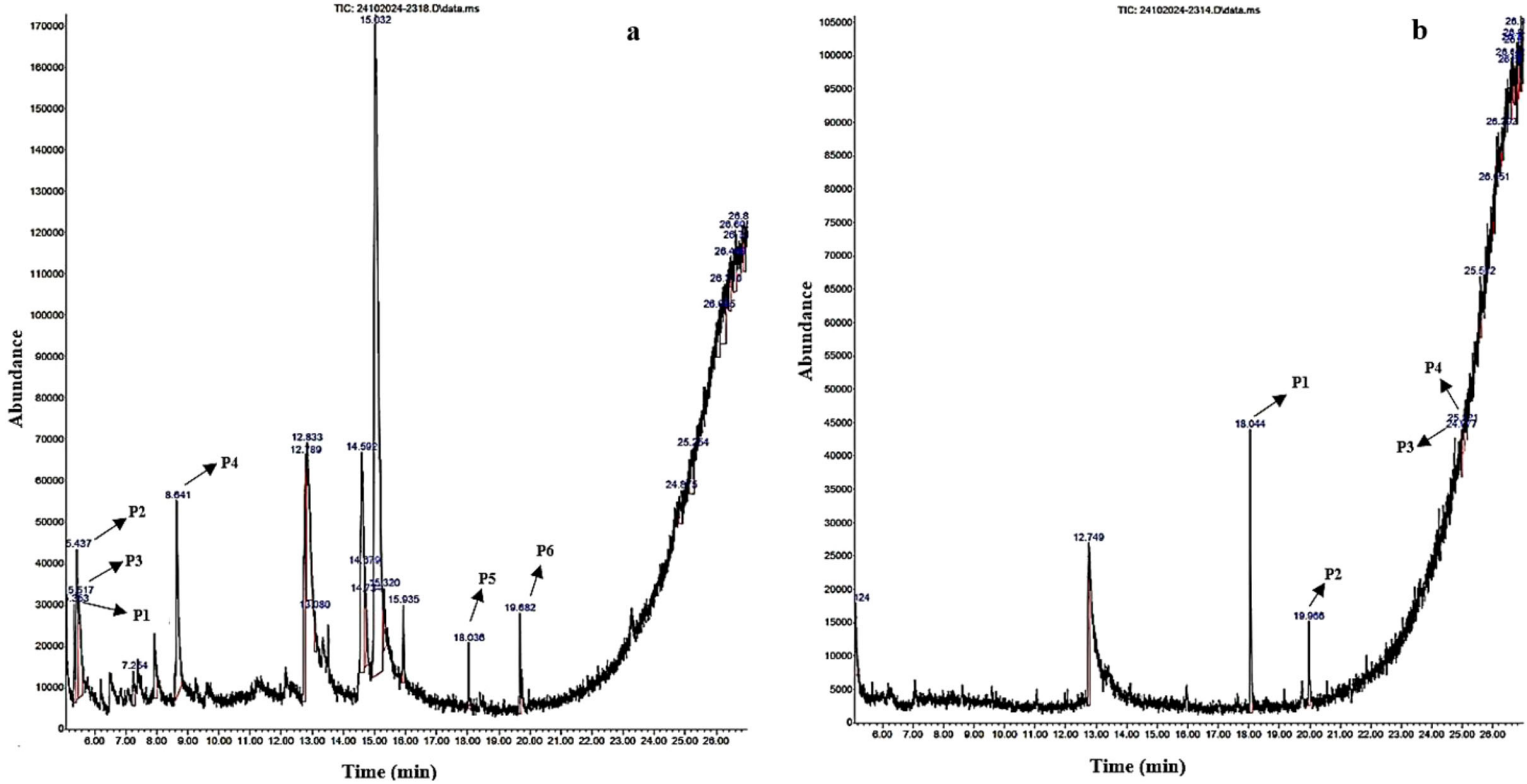
GC-MS chromatographic depiction of the bioactive metabolites of unelicited **(A)** and elicited **(B)** *Gardenia jasminoides* suspension cultures from 0.5 mg/L kinetin (KT) and 1 mg/L 2,4-dichlorophenoxyacetic acid (2,4-D) on Murashige and Skoog (MS) media at 36 h. The *arrows* highlight prominent peaks (*P1*–*P6*) relating to plant-derived bioactive metabolites.

**Figure 2:**
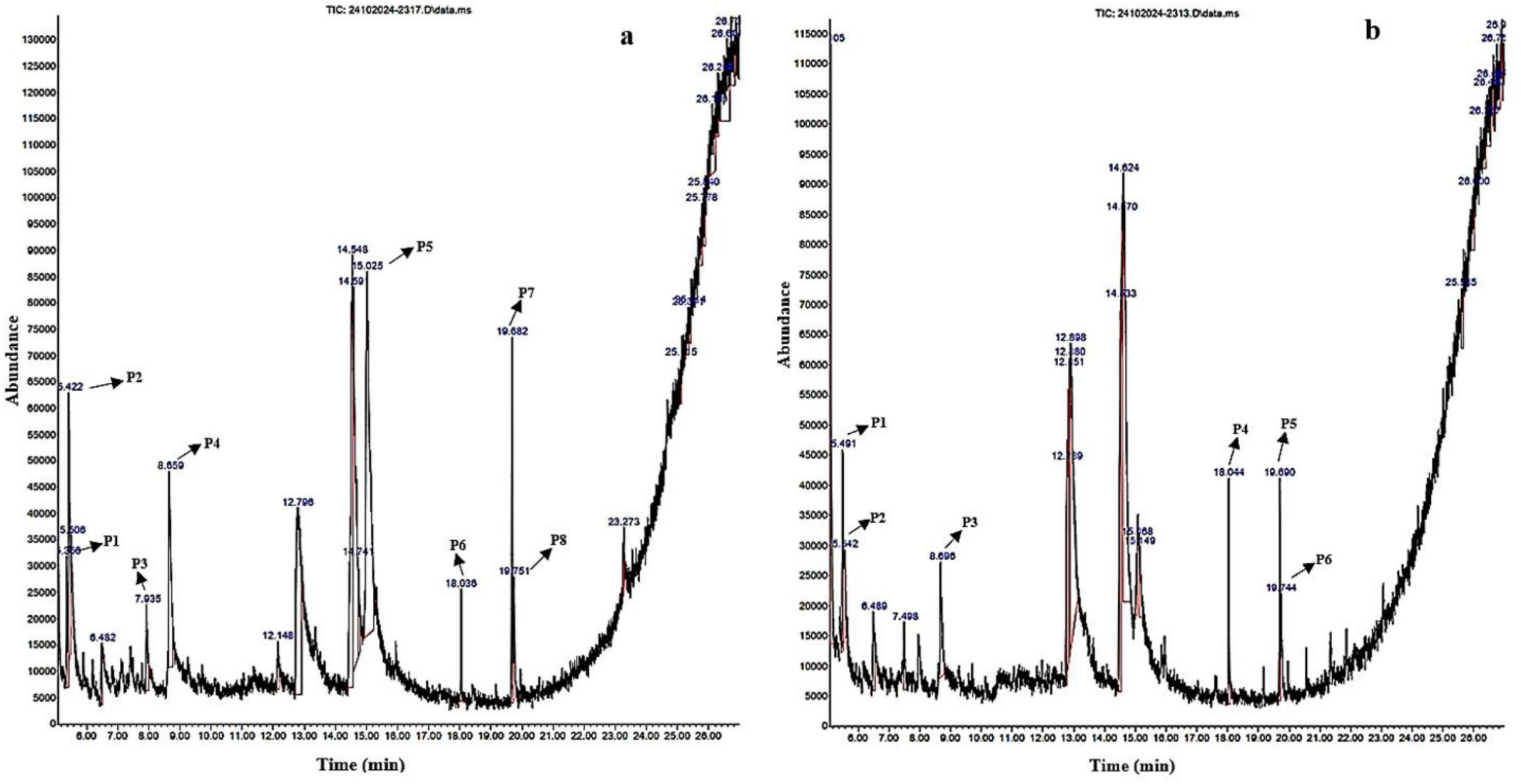
GC-MS chromatographic depiction of the bioactive metabolites of unelicited **(A)** and elicited **(B)** *Gardenia jasminoides* suspension cultures from 0.5 mg/L kinetin (KT) and 1 mg/L 2,4-dichlorophenoxyacetic acid (2,4-D) on Murashige and Skoog (MS) media at 120 h. The *arrows* highlight prominent peaks (*P1*–*P8*) relating to plant-derived bioactive metabolites.

**Figure 3:**
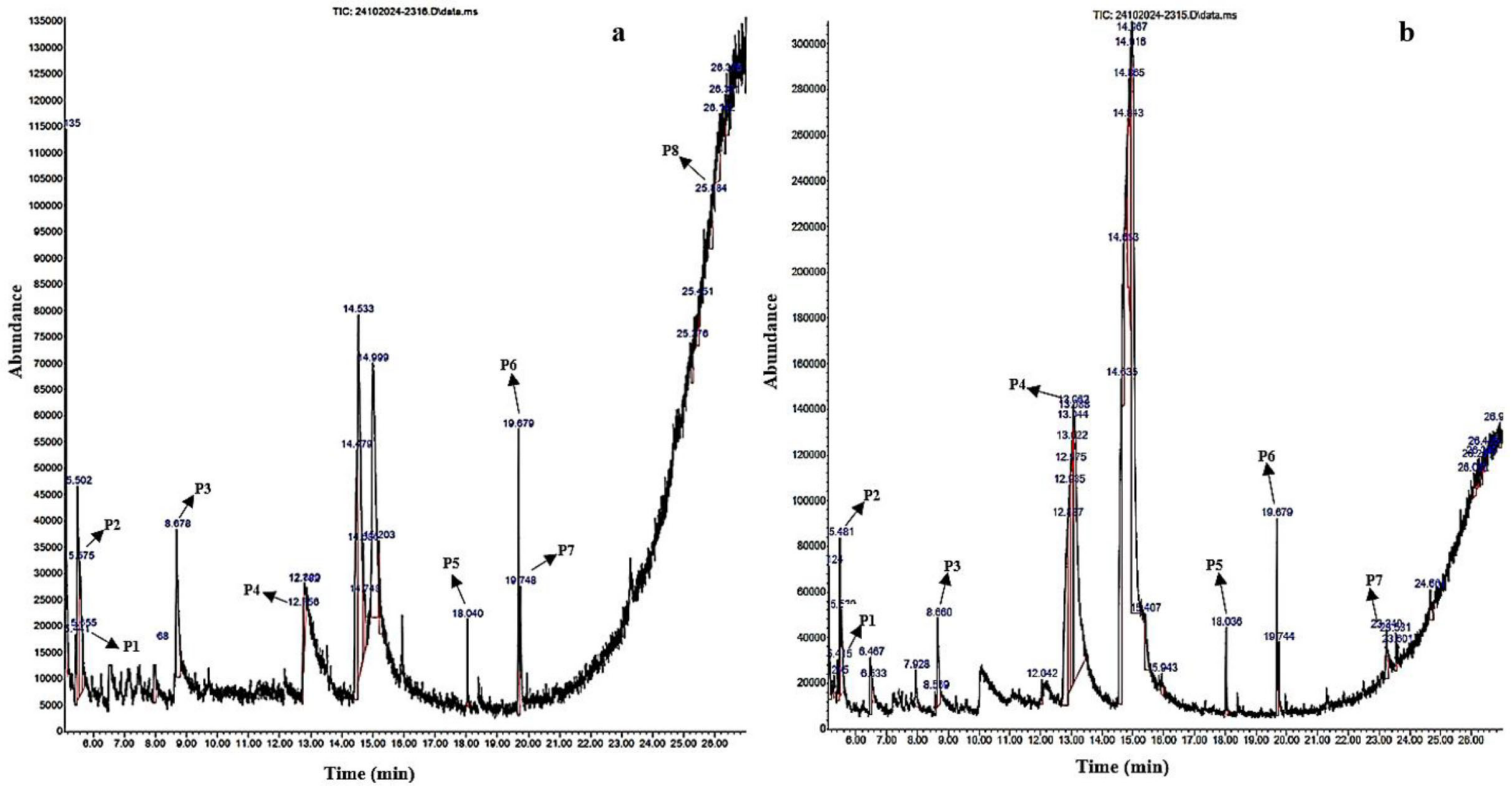
GC-MS chromatographic depiction of the bioactive metabolites of unelicited **(A)** and elicited **(B)** *Gardenia jasminoides* suspension cultures from 0.5 mg/L kinetin (KT) and 1 mg/L 2,4-dichlorophenoxyacetic acid (2,4-D) on Murashige and Skoog (MS) media at 144 h. The *arrows* highlight prominent peaks (*P1*–*P8*) relating to plant-derived bioactive metabolites.

Comparative metabolite profiling was performed using GC-MS on the methanolic extracts from both unelicited and CH-elicited *G. jasminoides* suspension cultures collected under optimized elicitation conditions corresponding to peak metabolite accumulation. Specifically, samples treated with 80 mg/L CH at 36 h (terpenoids) and 40 mg/L CH at 120 h (flavonoids) and 144 h (phenols) were analyzed as these conditions exhibited maximal accumulation of the respective metabolite classes (**Tables 3**–**5**; **Figures 1**–**3**). The curation of bioactive metabolites was confined to compounds of plant origin, with robust literature support attesting to their pharmacological efficacy. Distinct bioactive metabolites have been detected in CH-elicited suspension cultures, which were not expressed in unelicited suspension cultures. This distinction arises because CH can function as a potent biotic elicitor, triggering specific intracellular signaling cascades that upregulate dormant biosynthetic genes. These specialized metabolites are resultants of the plant defense mechanism against stress. Therefore, these bioactive metabolites were undetectable under less stress conditions in field-derived tissues and unelicited *in vitro* cultures. Moreover, bioactive metabolites synthesized through genomic activation in CH-elicited cell suspension cultures present pharmacological efficacy comparable to those extracted from naturally occurring plant tissues, as presented in **Table 6**.

**Table 3:** Comparative analysis of putatively identified bioactive metabolites in unelicited and chitosan (CH)-elicited *Gardenia jasminoides* suspension cultures at 36 h via GC-MS profiling.

S. no.	Control suspension culture at 36 h				80 mg/L CH at 36 h			
	Peak. no.	RT	Metabolite name and property	Peak area (%)	Peak. no.	RT	Metabolite name and property	Peak area (%)
Similar bioactive metabolites								
1	P5	18.036	Hexadecanoic acid, methyl ester (MF: C_17_H_34_O_2_; MW: 270.5)	0.73	P1	18.044	Hexadecanoic acid, methyl ester (MF: C_17_H_34_O_2_; MW: 270.5)	28.69
Dissimilar bioactive metabolites								
1	P1	5.353	Butyrolactone (MF: C_4_H_6_O_2_; MW: 86.09)	1.77	P2	19.966	Methyl 8-methyl-decanoate (MF: C_12_H_24_O_2_; MW: 200.32)	8.32
2	P2	5.437	Azetidine, 2-methyl- (MF: C_4_H_9_N; MW: 71.12)	3.01	P3	24.977	Benzo[*h*]quinoline, 2,4-dimethyl- (MF: C_15_H_13_N; MW: 207.27)	2.58
3	P3	5.517	4,5-Dihydro-2-methylimidazole-4-one (MF: C_4_H_6_N_2_O; MW: 98.1)	3.57	P4	25.021	(*E*)-3-(3,4-dimethoxyphenyl)-prop-2-enamide (MF: C_11_H_13_NO_3_; MW: 207.23)	3.17
4	P4	8.641	4*H*-Pyran-4-one, 2,3-dihydro-3,5-dihydroxy-6-methyl- (MF: C_6_H_8_O_4_; MW: 144.12)	6.05		–	–	–
5	P6	19.682	9,12-Octadecadienoic acid (*Z*,*Z*)-, methyl ester (MF: C_19_H_34_O_2_; MW: 294.5)	1.29		–	–	–

*MF*, molecular formula; *MW*, molecular weight; *RT*, retention time.

**Table 4:** Comparative analysis of putatively identified bioactive metabolites in unelicited and chitosan (CH)-elicited *Gardenia jasminoides* suspension cultures at 120 h via GC-MS profiling.

S. no.	Control suspension culture at 120 h				40 mg/L CH at 120 h			
	Peak no.	RT	Metabolite name and property	Peak area (%)	Peak no.	RT	Metabolite name and property	Peak area (%)
Similar bioactive metabolites								
1	P2	5.422	Azetidine, 2-methyl- (MF: C_4_H_9_N; MW: 71.12)	5.07	P1	5.491	Azetidine, 1-methyl- (MF: C_4_H_9_N; MW: 71.12)	3.50
2	P4	8.659	4*H*-Pyran-4-one, 2,3-dihydro-3,5-dihydroxy-6-methyl- (MF: C_6_H_8_O_4_; MW: 144.12)	5.79	P3	8.696	4*H*-Pyran-4-one, 2,3-dihydro-3,5-dihydroxy-6-methyl- (MF: C_6_H_8_O_4_; MW: 144.12)	3.32
3	P6	18.036	Hexadecanoic acid, methyl ester (MF: C_17_H_34_O_2_; MW: 270.5)	1.20	P4	18.044	Hexadecanoic acid, methyl ester (MF: C_17_H_34_O_2_; MW: 270.5)	2.72
4	P7	19.682	9,12-Octadecadienoic acid, methyl ester (MF: C_19_H_34_O_2_; MW: 294.46)	4.01	P5	19.690	9,12-Octadecadienoic acid (*Z*,*Z*)-, methyl ester (MF: C_19_H_34_O_2_; MW: 294.5)	2.61
Dissimilar bioactive metabolites								
1	P1	5.356	Butyrolactone (MF: C_4_H_6_O_2_; MW: 86.09)	1.99	P2	5.542	Cyclopentanone, 2-methyl- (MF: C_6_H_10_O; MW: 98.14)	2.43
2	P3	7.935	1-Pentanol, 4-amino- (MF: C_5_H_13_NO; MW: 103.16)	1.45	P6	19.744	*trans*-13-Octadecenoic acid, methyl ester (MF: C_19_H_36_O_2_; MW: 296.48)	2.06
3	P5	15.025	Octanoic acid (MF: C_8_H_16_O_2_; MW: 144.21)	20.20		–	–	–
4	P8	19.751	9,12,15-Octadecatrienoic acid, (*Z*,*Z*,*Z*)- (MF: C_18_H_30_O_2_; MW: 278.4)	1.78		–	–	–

*MF*, molecular formula; *MW*, molecular weight; *RT*, retention time.

**Table 5:** Comparative analysis of putatively identified bioactive metabolites in unelicited and chitosan (CH)-elicited *Gardenia jasminoides* suspension cultures at 144 h via GC-MS profiling.

S. no.	Control suspension culture at 144 h				40 mg/L CH at 144 h			
	Peak. no.	RT	Metabolite name and property	Peak area (%)	Peak. no.	RT	Metabolite name and property	Peak area (%)
Similar bioactive metabolites								
1	P1	5.441	Butyrolactone (MF: C_4_H_6_O_2_; MW: 86.09)	1.37	P1	5.415	Butyrolactone (MF: C_4_H_6_O_2_; MW: 86.09)	0.42
2	P3	8.678	4*H*-Pyran-4-one, 2,3-dihydro-3,5-dihydroxy-6-methyl- (MF: C_6_H_8_O_4_; MW: 144.12)	6.12	P3	8.660	4*H*-Pyran-4-one, 2,3-dihydro-3,5-dihydroxy-6-methyl- (MF: C_6_H_8_O_4_; MW: 144.12)	1.81
3	P5	18.040	Hexadecanoic acid, methyl ester (MF: C_17_H_34_O_2_; MW: 270.5)	1.28	P5	18.036	Hexadecanoic acid, methyl ester (MF: C_17_H_34_O_2_; MW: 270.5)	0.78
4	P6	19.679	9,12-Octadecadienoic acid (*Z*,*Z*)-, methyl ester (MF: C_19_H_34_O_2_; MW: 294.5)	4.35	P6	19.679	9,12-Octadecadienoic acid (*Z*,*Z*)-, methyl ester (MF: C_19_H_34_O_2_; MW: 294.5)	1.88
Dissimilar bioactive metabolites								
1	P2	5.575	Pyrrolidine (MF: C_4_H_9_N; MW: 71.12)	4.45	P2	5.481	Azetidine, 1-methyl- (MF: C_4_H_9_N; MW: 71.12)	2.09
2	P4	12.756	2-(3-Methylguanidino) ethanol (MF: C_4_H_11_N_3_O; MW: 117.15)	1.34	P4	13.062	N1-(4-hydroxybutyl)-N3-methylguanidine acetate (MF: C_9_H_21_N_3_O_4_; MW: 235.29)	3.05
3	P7	19.748	Methyl 8,11,14-heptadecatrienoate (MF: C_18_H_30_O_2_; MW: 278.4)	2.13	P7	23.240	Benzenamine, 2-(cyclopropylmethyl)-4,5-dimethoxy- (MF: C_12_H_17_NO_2_; MW: 207.27)	1.16
4	P8	25.884	Benzo[*h*]quinoline, 2,4-dimethyl- (MF: C_15_H_13_N; MW: 207.27)	1.47		–	–	–

*MF*, molecular formula; *MW*, molecular weight; *RT*, retention time.

**Table 6:** Pearson’s correlation matrix of GC-MS identified bioactive metabolites and their relative abundance in the control and chitosan (CH)-elicited *Gardenia jasminoides* cell cultures.

Bioactive metabolite	1	2	3	4	5	6	7
C36	−0.43						
C120	−0.12	−0.02					
C144	−0.12	0.21	0.08				
CH36	−0.39	−0.17	−0.08	−0.08			
CH120	−0.469*	0.14	0.11	0.4	0.31		
CH144	0.15	0.07	0.06	0.23	−0.18	0.35	

*N* = 19.

*C36*, control at 36 h; *C120*, control at 120 h; *C144*, control at 144 h; *CH36*, CH-elicited cell cultures at 36 h; *CH120*, CH-elicited cell cultures at 120 h; *CH144*, CH-elicited cell cultures at 144 h

*Correlation significant at the *p* < 0.05 level (two-tailed)

**Figure 4:**
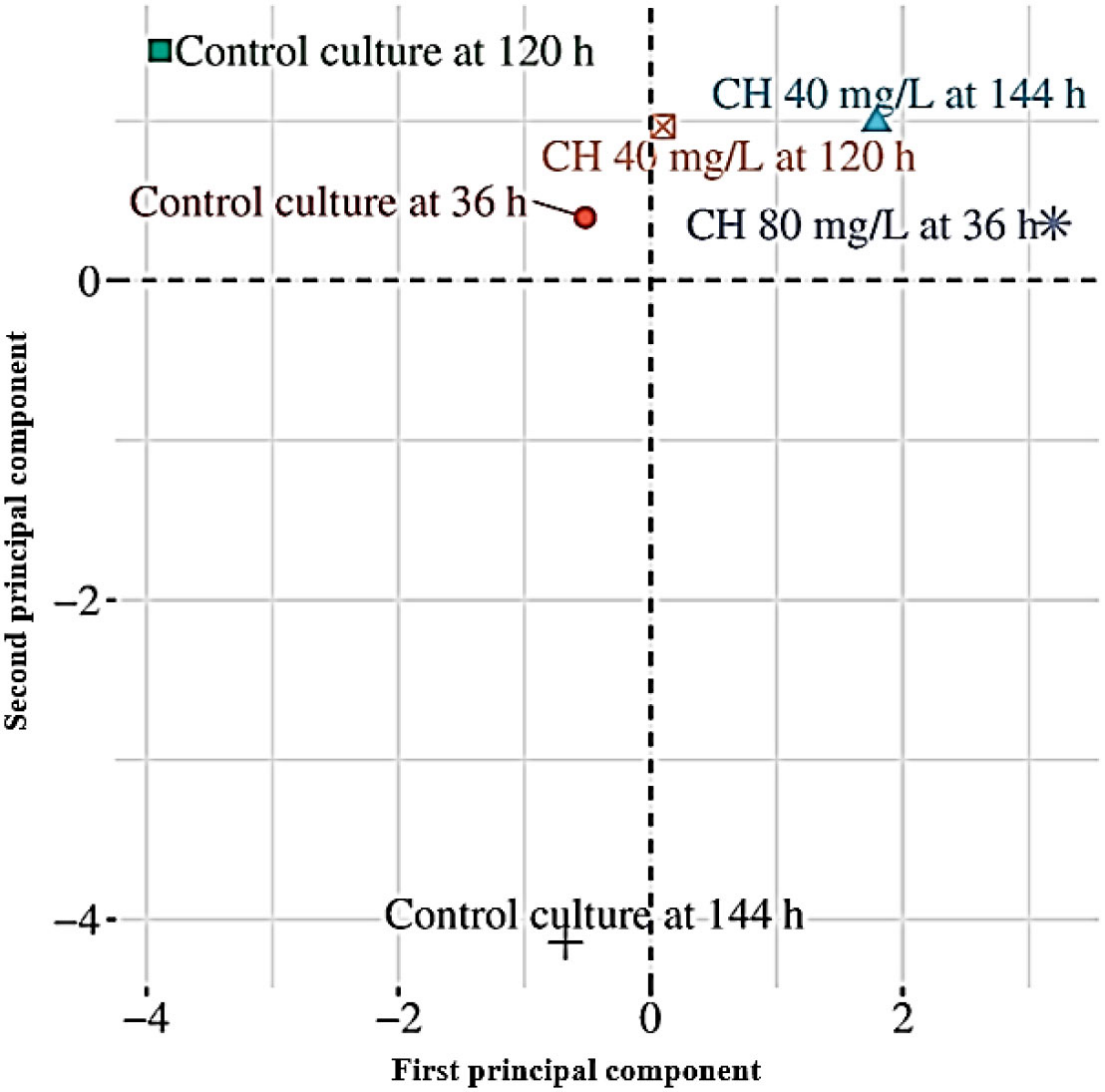
Principal component analysis (PCA) score plot of the bioactive metabolites determined by GC-MS in unelicited (control) and elicited suspension cultures of *Gardenia jasminoides.*.

To evaluate the captured metabolic variance, PCA was performed, with the first two components, PC1 and PC2, accounting for 32.6% and 23.8% of the total variance, respectively (**Figure 4**). The robustness of the PCA model was validated by a cumulative explained variance of 56.4%, representing a significant portion of metabolic diversity. The first principal component (PC1) was predominantly influenced by variations in several bioactive metabolites, including 4,5-dihydro-2-methylimidazole-4-one; hexadecanoic acid methyl ester; 4*H*-pyran-4-one, 2,3-dihydro-3,5-dihydroxy; 9,12-octadecadienoic acid (*Z*,*Z*)- methyl ester; (*E*)-3-(3,4-dimethoxyphenyl)-prop-2-enamide; and benzo[*h*]quinoline, 2,4-dimethyl. These bioactive metabolites displayed noticeably higher abundance in the CH-treated cultures, particularly at 80 mg/L CH at 36 h and 40 mg/L CH at 120 h, which explains their high positive loading contribution to PC1 and the distinct clustering of these treatments in the PCA score plot. The second principal component (PC2) was largely driven by the differential accumulation of octanoic acid, 9,12,15-octadecatrienoic acid, cyclopentanone, 2-methyl-, pyrrolidine, butyrolactone, and 2-(3-methylguanidino) ethanol. These bioactive compounds showed prominent temporal variations, particularly between the control cultures at 36 and 144 h, indicating that the culture duration had a critical influence on their metabolic expression, independent of elicitation. Together, these metabolite-specific contributions validate the separation observed among treatments in the PCA score plot and confirm 9,12-octadecadienoic acid methyl ester, hexadecanoic acid methyl ester, and nitrogen-containing heterocyclic compounds as key determinants of the CH-mediated metabolic shifts in the cell cultures. Furthermore, the bioactive constituents identified in the CH-elicited suspension culture extracts displayed diverse therapeutic and functional properties, such as hepatoprotective, antioxidative, antifungal, pesticidal, anti-inflammatory, and anti-arthritic activities (Gupta et al., 2023). The chemical identities of the primary metabolites were rigorously validated through a comparative analysis of their mass fragmentation patterns against the NIST20 spectral library, ensuring high-probability matches (similarity index >90%). Our findings are consistent with related research, where hexadecanoic acid methyl ester (palmitic acid ester), 9,12-octadecadienoic acid methyl ester (linoleic acid ester), and various nitrogen-containing heterocyclic compounds were identified in the leaves, shoots, and suspension cultures of *G. jasminoides* (Krasteva et al., 2022). This alignment between our experimental mass spectral data and the reported metabolic profiles of different tissue types confirms the chemical stability of the biosynthetic pathways in our elicited cell lines.

**Figure 5:**
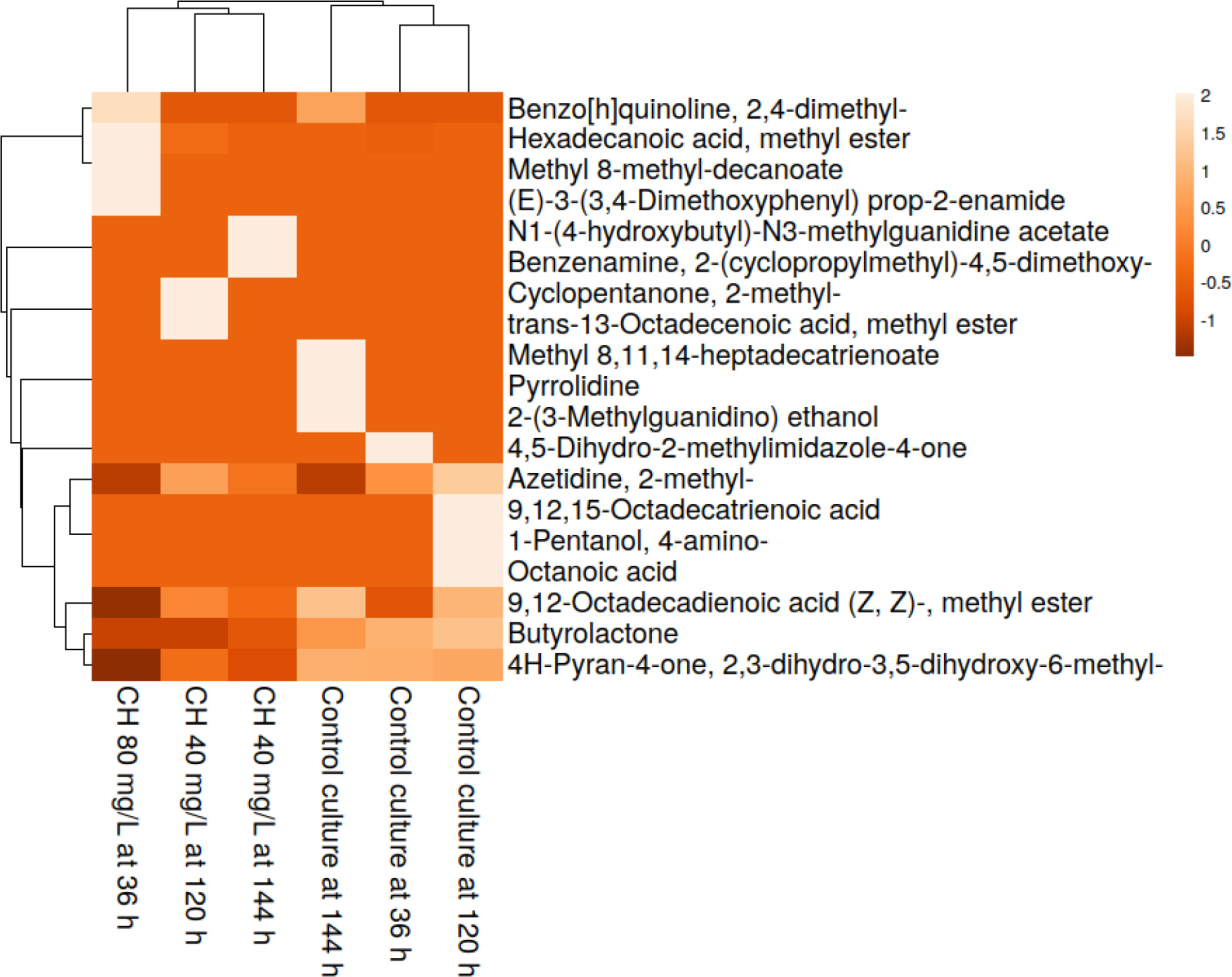
Hierarchical clustering heatmap showing the profile variations of the bioactive metabolites in the unelicited and chitosan (CH)-elicited *Gardenia jasminoides* suspension cultures as determined by GC-MS. The *columns* show individual sample extracts (control: 36, 120, and 144 h; CH: 80 mg/L at 36 h and 40 mg/L at 120 and 144 h). *Rows* represent metabolites, ordered by relative abundance, and display scaled intensity values (−1 to +2) represented in *shades of orange* (ascending from low to high accumulation).

To visually represent the metabolic profiles, a hierarchical heatmap (**Figure 5**) was generated using Euclidean distance and Ward’s linkage to ensure robust clustering, comparing the relative abundance of the key bioactive metabolites between the unelicited and the CH-elicited (40 and 80 mg/L) *G. jasminoides* suspension cultures. Hierarchical analysis of the extracts showed clustering between the unelicited and CH-elicited cultures, suggesting a significant function for CH elicitation. Sub-clustering analysis demonstrated similar outcomes, in which samples with CH also aligned based on the time points. The findings showed that the later stages (120 and 144 h) were grouped separately, reflecting the time-dependent modification of the metabolites.

Pearson’s correlation analysis was performed to establish a functional link between metabolic shifts and cellular potency (**Table 6**). Notably, the CH 120-h treatment, which also exhibited maximal total flavonoid content (TFC) values (23.63 ± 0.26 mg QE/g), also demonstrated a statistically significant inverse correlation in metabolite distribution (*r* = −0.469, *p* = 0.043). This indicates that the peak accumulation of flavonoids coincides with a highly organized volatile profile, where the most abundant elicited constituents, identified as nitrogenous compounds and fatty acid esters, are non-randomly concentrated. The convergence of peak TFC and a significant metabolic signature at 120 h confirmed this interval as the optimal window for elicitor-mediated secondary metabolism.

To establish a discernible link between the chemical profile and the observed biological activities, **Table 7** presents the metabolites that were induced *de novo* in the elicited cultures and were absent in the control cultures. All identified peaks were matched against the NIST20 library and verified in the existing literature. The screened metabolites (**Table 7**) were selected based on their biogenic origin and documented pharmacological properties.

**Table 7:** List of putatively identified bioactive metabolites observed through GC-MS analysis in chitosan (CH)-elicited *Gardenia jasminoides* suspension cultures.

S. no.	Metabolite name	Pharmacological effect	Reference
1	N1-(4-hydroxybutyl)-N3-methylguanidine acetate	Demonstrated inhibitory effects on microbial growth under *in vivo* and *in vitro* conditions	Sánchez-Hernández et al., 2023
2	Methyl 8-methyl-decanoate	Inferential evidence from Ayurvedic Kashaya formulations points to potential anti-inflammatory, antioxidant, and antimicrobial effects	LS and Anand, 2024
3	Benzenamine, 2-(cyclopropylmethyl)-4,5-dimethoxy-	Structurally related to serotonergic agents and suggested to possess potential 5-HT receptor–ligand activity (neuropharmacology research)	Pigott et al., 2012; Kolaczynska et al., 2019
4	(*E*)-3-(3,4-Dimethoxyphenyl) prop-2-enamide	A cinnamamide derivative with putative anti-inflammatory and antioxidant effects, inferred from the pharmacological profiles of structurally related 3,4-dimethoxy cinnamic acid derivatives	Bandaru and Kuchana, 2024
5	*trans*-13-Octadecenoic acid, methyl ester	Exhibit antioxidant and antimicrobial effects	Awonyemi et al., 2020

## 4 Discussion

The radical scavenging activity reported here is intrinsically linked to the chemical composition of the extract. Specifically, our previous analysis revealed a total phenol content (TPC) of 10.65 ± 0.05 mg GAE/g and TFC of 23.63 ± 0.26 mg QE/g (Kumar and Parasurama, 2024a). The predominance of flavonoids, which are excellent hydrogen atom donors, explains the efficiency of the extract in neutralizing DPPH radicals. This strong stoichiometric relationship between the phenol or flavonoid concentration and the radical scavenging capacity provides a comprehensive validation of the antioxidant potential, aligning with the established literature where TPC and TFC are primary predictors of DPPH inhibition in *G. jasminoides*. The high phenol and flavonoid contents suggest a chemical profile dominated by potent scavengers, making the DPPH assay a highly sensitive and appropriate tool for measuring their bioactivity. The extracts from both non-elicited and CH-elicited suspension cultures possessed pronounced antioxidative activity with dose-dependent enhancement in DPPH inhibition activity. A dose-dependent increase in DPPH scavenging activity was also assessed in our previous work on the antioxidative effects of the extractions from *G. jasminoides in vitro* callus and suspension cultures (Kumar and Parasurama, 2024a). Similar results were recorded, in which treatment of CH elicitation in the *Linum usitatissimum* L. suspension cultures enhanced their antioxidant capacity, with a 1.35-fold increase compared with the untreated (control) suspension culture (Ahmad et al., 2019). Antioxidant capacity is the ability to mitigate the production of reactive oxygen species, which are harmful and responsible for oxidative stress in the cellular constituents (i.e., proteins, membrane lipids, and nucleic acids) (Gholamian et al., 2017). CH, a potent biotic elicitor, critically upregulates the expression of the defense-related genes to improve secondary metabolites and reinforce the antioxidant activity (Gai et al., 2019). Moreover, a similar study reported that CH application to *Ginkgo biloba* L. callus cultures, which had high total phenols and flavonoids, showed the greatest antioxidant activity (Elateeq et al., 2021). CH elicitation in *in vitro* cultures caused total phenol and flavonoid enhancement, corresponding to a positive relationship with improved antioxidant potency. In addition, the findings emphasize that CH-mediated elicitation plays a crucial function in the upregulation of secondary metabolic pathways, resulting in enhanced antioxidant potency. The enhanced antioxidant activity in the CH-elicited extracts can also be attributed to the induction of cinnamamide derivatives, such as (*E*)-3-(3,4-dimethoxyphenyl)-prop-2-enamide. Structurally, the presence of methoxyl groups on the benzene ring facilitates electron donation, which is a key mechanism for neutralizing DPPH radicals. Furthermore, the co-occurrence of these bioactive metabolites with upregulated phenolic acids suggests a cumulative antioxidant response triggered by CH elicitation.

*In vitro G. jasminoides* cells may have high levels of antimicrobial activity mainly due to the synthesis of secondary metabolites, which may play an important role in the suppression of microbial growth (Farzinebrahimi et al., 2014). The inhibitory effect of 40 mg/L CH was more significant than that of 80 mg/L CH, indicating that the total phenol (40 mg/L CH at 144 h) and flavonoid (40 mg/L CH at120 h) accumulation might play a role in their contribution to antibacterial activity (Luo et al., 2021; Kumar and Parasurama, 2025). This finding may indicate a significant association of higher phenol and flavonoid contents with bacterial inhibition (Luo et al., 2021). While the commercial antibiotic erythromycin exhibited superior antibacterial activity, the elicited *G. jasminoides* suspension culture extracts showed only modest antimicrobial effects, with the inhibition zones remaining below 7 mm for all tested strains. Despite the enhanced total phenolic and flavonoid contents and the strong radical scavenging capacity, the limited antibacterial efficacy suggests that the metabolite profile is primarily oriented toward oxidative stress mitigation rather than effective disruption of the bacterial cell structures or the metabolic pathways. Moreover, although CH elicitation enhances phenol and flavonoid biosynthesis, these compounds may lack the specific structural features required for potent antibacterial activity at the tested concentrations. This indicates that the extracts are better characterized by their antioxidant potential than by their antimicrobial strength. Thus far, only one earlier report concerning the antibacterial potency of *G. jasminoides in vitro* callus cultures against the four specified bacterial microorganisms indicates that callus supplemented with 1-naphthaleneacetic acid (NAA) exhibited inhibitory efficacy against *E. coli* and *B. subtilis*, while no antibacterial effects were observed against *S. aureus* and *P. aeruginosa* (Farzinebrahimi et al., 2014). Comparable reports on the antibacterial efficacy of *in vitro* callus and suspension cultures of *Cleome rosea* Vahl and *Thevetia peruviana* against *S. aureus*, *E. coli*, and *B. subtilis* have been documented (Simões et al., 2012; Echeverri et al., 2019).

GC-MS analysis revealed a distinct metabolic shift in *G. jasminoides* suspension cultures following CH elicitation. The CH-elicited cultures enhanced the concentrations of 9,12-octadecadienoic acid methyl ester, hexadecanoic acid methyl ester, 9,12,15-octadecatrienoic acid, octanoic acid, pyrrolidine, azetidine, 2-methyl-, and 2-(3-methylguanidino) ethanol, while the unelicited cultures retained more primary metabolite values for simple sugars and structural compounds. Several bioactive compounds were exclusively detected in the CH-elicited samples, while they remained below the analytical detection limit in the unelicited control groups. The presence of these metabolites suggests that CH elicitation induced rerouting of the metabolic flux toward the defense-related secondary metabolite pathways, leading to the accumulation of bioactive constituents in *G. jasminoides* suspension cultures that are not discernible under basal conditions. While this qualitative profiling highlights the induction of a specialized metabolome, further quantitative studies are warranted to determine the absolute concentrations and precise fold changes of these induced compounds.

The synchronization between the maximal TFC and the significant Pearson’s correlation at CH120 highlights profound metabolic reprogramming in *G. jasminoides* cell cultures. CH elicitation appears to trigger a pulse of secondary metabolism that is both chemically diverse and quantitatively superior to TFC. The inverse correlation (*r* = −0.469) suggests that at the 120-h peak, cellular resources are heavily partitioned into a specific suite of high-abundance flavonoids and volatile esters. This is further supported by the moderate correlation observed between CH at 120 h and the late-stage control at 144 h (*r* = 0.401, *p* = 0.089), reinforcing the hypothesis that elicitation effectively accelerates the maturation of the defensive chemical matrix. Consequently, the functional properties of *G. jasminoides* cell culture are not a result of general stress, but a directed biosynthetic response that maximizes the yield of specialized metabolites precisely at the 120-h mark.

The observation of peak TFC at 120 h, followed by peak TPC at 144 h, provides a physiological proxy for the sequential activation of the phenylpropanoid pathway (Kumar and Parasurama, 2024a). This progression is consistent with the metabolic flux typical of CH-elicited systems, where early response genes (chalcone synthase) reach peak expression before the stabilization of the total phenolic sink. These findings suggest that CH120 represents the optimal window for specialized metabolite diversity, while CH144 marks the peak time for quantitative phenolic yield. While these compounds have been previously reported in wild plants or related botanical species, their specific induction in this study suggests that CH triggers latent biosynthetic pathways in the *G. jasminoides* cell line.

## 5 Conclusions

The present study demonstrates that the strategic application of CH as a biotic elicitor effectively modulates the secondary metabolome of *G. jasminoides* cell suspension culture. Optimization of the treatment parameters revealed that specific concentrations and exposure times are critical for maximizing the biosynthesis of specialized metabolites. Notably, 40 mg/L CH significantly enhanced the accumulation of phenolic and flavonoid compounds, which directly correlated with a superior antioxidant capacity compared with the non-elicited controls. GC-MS profiling confirmed that elicitation triggered the *de novo* expression of bioactive metabolites not detected under basal conditions, suggesting the activation of latent, defense-related pathways. While the antimicrobial response remained modest, likely due to the metabolic prioritization of antioxidant-related phenylpropanoid derivatives, these findings validate the use of cell cultures as a sustainable platform for metabolic enrichment. These results provide a robust baseline for the targeted induction and subsequent isolation of therapeutic biomolecules from *G. jasminoides*, highlighting the potential of elicitation in accelerating the discovery of stress-induced natural products.

## Data Availability

The original contributions presented in the study are included in the article/supplementary material. Further inquiries can be directed to the corresponding author.
